# Large-scale sampling of *C. elegans* on *E. coli* HT115 RNAi-compatible bacteria

**DOI:** 10.17912/micropub.biology.000692

**Published:** 2023-01-09

**Authors:** Rose Boelen, Liesbet Temmerman

**Affiliations:** 1 University of Leuven (KU Leuven)

## Abstract

As opposed to standard culturing, growing large numbers of *C. elegans* worms on Nematode Growth Medium-based RNA interference (RNAi) plates requires multiple transferring steps to prevent starvation, which increase handling time and reduce practical efficiency. We here provide an optimized method to grow four times more worms in RNAi conditions, thus saving on required resources and handling steps.

**Figure 1.**
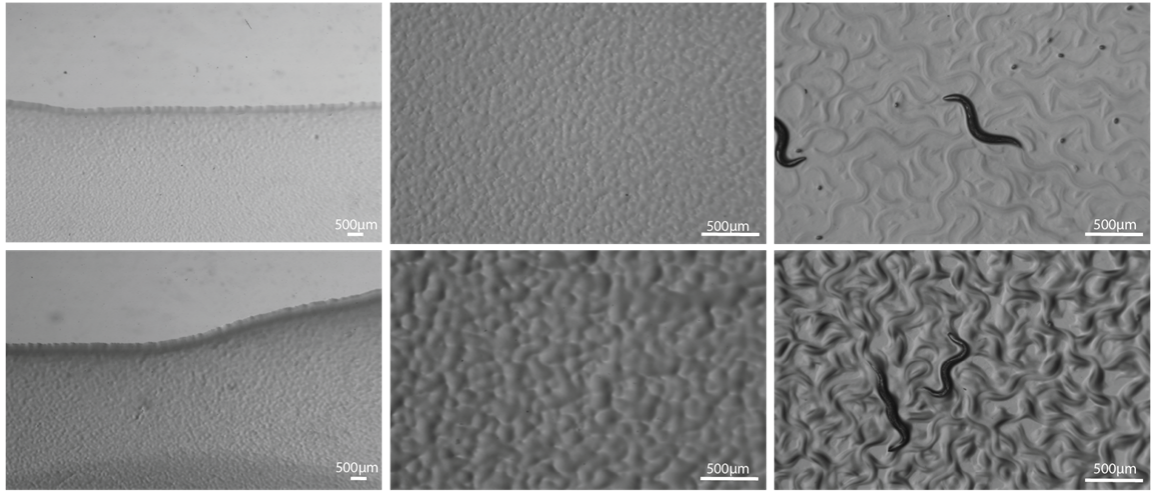
**Bacterial growth of HT115 (DE3) carrying the L4440 empty vector on Nematode Growth Medium** (top row panels) **versus Nutrient Agar medium** (bottom panels), each containing 1 mM IPTG and 50 µg/ml ampicillin. Left: overview of bacterial layer, seeded at OD _600_ =1.8 and grown at 37 °C for 24 hours. Middle: detail of the lawn, which remains thin for Nematode Growth Medium (top) but forms a thick layer on Nutrient Agar (bottom). Right: the bacterial layer after maintaining an adult worm culture for 24 hours (2000 worms on 90 mm Petri dish).

## Description

Sample preparation for *Caenorhabditis elegans* research sometimes requires rearing multiple thousands of animals. While this is rather straightforward under standard conditions, it tends to be a challenge when RNAi by feeding (Dudley & Goldstein, 2005) is desired, mainly due to the food source limiting the achievable culture density.

Inducing RNAi by feeding is a commonly used technique in *C. elegans* , with the Ahringer and Vidal RNAi libraries as useful resources (Timmons & Fire, 1998, Kamath *et al* ., 2003; Rual *et al* ., 2004). Both libraries use the *E. coli* strain HT115 (DE3), which contains an isopropyl beta-D-1-thigalactopyranoside (IPTG)-inducible T7 polymerase site to promote the production of double-stranded RNA (dsRNA). The standard protocol relies on Nematode Growth Medium (NGM) with IPTG and ampicillin/carbenicillin as seeding medium for the HT115 (DE3) layer, onto which worms can then be cultured (Kamath *et al* ., 2001; Ahringer, 2006). However, only a thin layer of bacteria will grow on these plates (figure 1, top row). When more than 1000 worms are plated in a standard 90 mm Petri dish, this therefore quickly leads to starvation and burrowing. When large numbers of worms are needed, experiments quickly become inefficient due to the many transferring steps that are needed to keep the animals fed. We here provide an optimized method for growing large amounts of *C. elegans* on HT115 (DE3), effectively limiting transferring steps and making it suitable for larger scale sampling.

The optimized protocol is based on three principles: concentrating the bacterial culture for a higher density, using Nutrient Agar plates with higher nutrient content to allow for better bacterial growth, and increasing the agar concentration to avoid burrowing of worms. By using different plates and optimized bacterial growth conditions, up to 2000 worms can be maintained per plate; this is 4 times more than on NGM RNAi plates. A similar larval lethality rate was observed on both Nutrient Agar RNAi and NGM RNAi plates after knockdown of *eef-2* (Fraser *et al* ., 2000) *,* verifying the RNAi efficacy of the optimized protocol. Overall, we provide a method for the community that allows for an increased number of worms to be cultured on *E. coli* HT115 (DE3) RNAi bacteria compared to previous protocols, reducing the number of required resources and handling steps.

## Methods

Nutrient agar plate preparation

The bacterial layer on NGM plates (supplemented with 1 mM IPTG (Thermo Scientific™) and 50 µg/ml ampicillin) remained thin (figure 1; top row). Even when seeding at high concentrations (OD _600_ =1.8) and increasing the amount of bacteria that were seeded onto the plate (up to 350 µl, *vs* our lab’s standard 150 µl for a 90 mm Petri dish), this still led to starvation and burrowing when more than a 1000 worms were plated. We therefore opted for the use of Nutrient Agar (Thermo Scientific™) plates (NA plates, also supplemented with 1 mM IPTG and 50 µg/ml ampicillin). This richer medium allows the bacteria to grow to a thick layer (figure 1; bottom row), providing sufficient food for the worms for several days. Our NA plate recipe relies on 2.5 % agar, which in contrast to lower (1.7 %) agar concentrations prevents burrowing after 2-3 days of culturing. Plates were always poured 2-3 days prior to seeding and stored at 20 °C in the dark.

*E. coli* HT115 (DE3) bacteria preparation

*E. coli* HT115 (DE3) (Ahringer/Vidal) were grown overnight in a shaking incubator in LB medium with 50 µg/ml ampicillin at 37 °C, as the standard RNAi protocol dictates (Ahringer, 2006). After 16 hours, IPTG was added to the medium (1 mM final concentration) and cultures were further incubated for exact two hours at 37 °C to induce dsRNA production. Prior to seeding, bacterial cultures were concentrated to an OD _600_ of 1.8 by spinning the culture for 6 min at 4000 *g* and resuspending the pellet in an appropriate volume of fresh LB medium. Plates were seeded with 350 µl of concentrated HT115 (DE3) and incubated at 37 °C for 24 hours to permit the growth of a bacterial lawn (figure 1; bottom row), after which they were stored in the dark until further use.

Worm preparation

N2 wild-types were grown on *E. coli* OP50 prior to synchronization. Worms were bleached and eggs were collected by standard procedures, after which they were left to hatch overnight to synchronize the population in L1 arrest (Stiernagle, 2006). We next reared worms on plates seeded with HT115 (DE3) control (empty vector) until the L4 stage (48 hours after plating L1 juveniles), after which they were collected and plated onto the experimental RNAi plates. Avoiding progeny was achieved through the use of 5-Fluoro-2'-deoxyuridine (FUdR), which was added to the experimental plates to an end concentration of 100 µM (Saul *et al* ., 2022). Worms were transferred once onto new RNAi plates (containing 100 µM of FUdR) after three days to avoid the risk of starvation, after which they were kept until day 14 of adulthood without extra transfer steps being required. The use of FUdR, which despite some side effects (Angeli *et al* ., 2013; McIntyre *et al* ., 2021; Wang *et al* ., 2019), is one of the most practical and popular means to induce sterility in *C. elegans* . Alternatives include use of sterile mutants or daily transfer of animals, which are less interesting for high throughput experiments under control conditions (including constant temperature) (Mei & Singson, 2020).

## Reagents

Strains: N2 (Bristol wild-type), available from the CGC (Minnesota, USA)

Chemicals: isopropyl beta-D-1-thigalactopyranoside (IPTG; 10725471, Thermo Scientific™), Nutrient Agar (Dehydrated; 10219252, Thermo Scientific™), 5-Fluoro-2'-deoxyuridine (FUdR; F0503, Sigma-Aldrich)

## References

[R1] Ahringer Julie (2006). WormBook.

[R2] Angeli S, Klang I, Sivapatham R, Mark K, Zucker D, Bhaumik D, Lithgow GJ, Andersen JK (2013). A DNA synthesis inhibitor is protective against proteotoxic stressors via modulation of fertility pathways in Caenorhabditis elegans.. Aging (Albany NY).

[R3] Dudley NR, Goldstein B (2005). RNA interference in Caenorhabditis elegans.. Methods Mol Biol.

[R4] Fraser AG, Kamath RS, Zipperlen P, Martinez-Campos M, Sohrmann M, Ahringer J (2000). Functional genomic analysis of C. elegans chromosome I by systematic RNA interference.. Nature.

[R5] Kamath RS, Fraser AG, Dong Y, Poulin G, Durbin R, Gotta M, Kanapin A, Le Bot N, Moreno S, Sohrmann M, Welchman DP, Zipperlen P, Ahringer J (2003). Systematic functional analysis of the Caenorhabditis elegans genome using RNAi.. Nature.

[R6] Kamath RS, Martinez-Campos M, Zipperlen P, Fraser AG, Ahringer J (2000). Effectiveness of specific RNA-mediated interference through ingested double-stranded RNA in Caenorhabditis elegans.. Genome Biol.

[R7] McIntyre G, Wright J, Wong HT, Lamendella R, Chan J (2021). Effects of FUdR on gene expression in the C. elegans bacterial diet OP50.. BMC Res Notes.

[R8] Mei X, Singson AW (2020). The molecular underpinnings of fertility: Genetic approaches in Caenorhabditis elegans.. Adv Genet (Hoboken).

[R9] Rual JF, Ceron J, Koreth J, Hao T, Nicot AS, Hirozane-Kishikawa T, Vandenhaute J, Orkin SH, Hill DE, van den Heuvel S, Vidal M (2004). Toward improving Caenorhabditis elegans phenome mapping with an ORFeome-based RNAi library.. Genome Res.

[R10] Saul N, Dhondt I, Kuokkanen M, Perola M, Verschuuren C, Wouters B, von Chrzanowski H, De Vos WH, Temmerman L, Luyten W, Zečić A, Loier T, Schmitz-Linneweber C, Braeckman BP (2022). Identification of healthspan-promoting genes in Caenorhabditis elegans based on a human GWAS study.. Biogerontology.

[R11] Stiernagle T (2006). Maintenance of C. elegans.. WormBook.

[R12] Timmons L, Fire A (1998). Specific interference by ingested dsRNA.. Nature.

[R13] Wang H, Zhao Y, Zhang Z (2019). Age-dependent effects of floxuridine (FUdR) on senescent pathology and mortality in the nematode Caenorhabditis elegans.. Biochem Biophys Res Commun.

